# Challenges and Solutions in Provision of Health Services to People With Disabilities During Mass Gatherings

**DOI:** 10.1002/puh2.70317

**Published:** 2026-08-10

**Authors:** Arezoo Dehghani, Fateme Behmenshpour, Sajjad Anami, Gholamreza Masoumi, Zahra Eskandari

**Affiliations:** ^1^ PhD of Health in Disasters and Emergencies, Emergency Medicine Management Research Center Health Management Research Institute, Iran University of Medical Sciences Tehran Iran; ^2^ Department of Nursing, School of Nursing Alborz University of Medical Sciences Karaj Iran; ^3^ Master of Urban Planning Shahr Quds Islamic Azad University Tehran Iran; ^4^ Professor of Emergency Medicine, Emergency Medicine Management Research Center Health Management Research Institute, Iran University of Medical Sciences Tehran Iran; ^5^ Department of Prehospital Emergencies, School of Nursing Alborz University of Medical Sciences Karaj Iran

**Keywords:** health services for disabled persons, mass gathering, people with disabilities, promotion of health

## Abstract

**Background:**

Although individuals with disabilities are more vulnerable and face higher risks of injury during mass gatherings, this population remains understudied. Their health may be at risk unless healthcare access barriers during large events are addressed. Therefore, this study aimed to identify the challenges that this at‐risk group encounters when trying to obtain healthcare during mass gatherings.

**Materials and Methods:**

A qualitative research study was carried out from May 6 to August 22, 2023. Semi‐structured interviews were conducted with 26 participants, which included individuals with disabilities, policymakers, managers, and specialists in disaster management. We employed purposive and snowball sampling techniques, continuing recruitment and data collection until theoretical saturation. The interviews reached saturation at this stage, with no new themes emerging. The gathered data were analyzed using both conventional content analysis and the Graneheim and Lundman approach.

**Results:**

On the basis of the findings, the identified challenges were categorized into two main groups: (1) challenges for managers and policymakers (including service provision, logistics, coordination, and planning); and (2) challenges for people with disabilities (including care support services, provision of infrastructure, and management of health services).

**Conclusions:**

In order to ensure equity in access to health services, this study highlighted two key policy strategies: (1) the ongoing and meaningful participation of people with disabilities in designing, planning, and implementation health programs, and (2) multi‐sectoral collaboration among health policymakers and relevant institutions to effectively integrate the needs of people with disabilities in to national and local mass gathering management programs.

## Graphical abstract

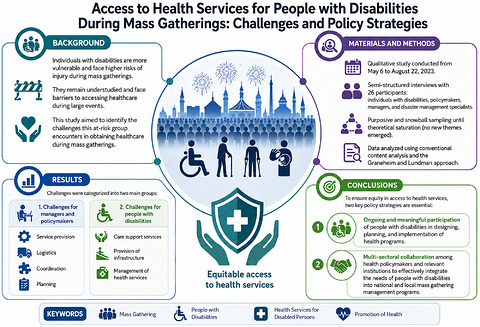

## 1 Introduction

According to the World Health Organization, mass gatherings are events where large crowds may strain public health resources [1]. They require careful planning [2, 3], but challenges such as crowd management, poor emergency preparedness, inadequate laws, budget shortfall, and insufficient training often hinder hazard control [1, 4].

The Arbaeen pilgrimage in Karbala attracts over 20 million participants annually [5, 6, 7]. Most pilgrims walk 80 km from Najaf to Karbala in 20–25 h [8]. Similar to other mass gatherings, overcrowding, poor hygiene, and inadequate health and sanitation practices increase infectious disease risks and health discomfort [5, 9].

People with disabilities are particularly vulnerable. Globally, 1.3 billion people (16%) have disability, a figure projected to double by the year 2030, with 80% in low‐ and middle‐income countries [10, 11]. In Iran, estimates range from 1% to 4% of the population [12]. Iran joined the UN Convention on the Rights of People with Disability in 2008, committing to key principles like accessibility, nondiscrimination, and inclusive participation [13].

These individuals possess distinct medical requirements, necessitating a greater level of care than is required for the general population, due to their specific conditions and the numerous barriers they face in obtaining care [11, 14]. Furthermore, due to inequalities within the laws, policies, and procedures themselves, people with disabilities often lack representation in decision‐making processes and do not meaningfully participate in relevant policymaking [15]_._ Consequently, they face discrimination enshrined in laws and policies across various sectors, including education, healthcare provision, and public administration [16].

Limited research addressed the challenges people with disabilities face in mass gatherings. Using the Arbeen pilgrimage as a case study, this study explores these challenges and offers recommendations.

## 2 Materials and Methods

### 2.1 Study Design

We used a qualitative inductive content analysis approach.

### 2.2 Study Participants and Sampling

The study included two groups: (1) people with disabilities who had participated in the Arbaeen walk at least once during the past 5 years, and (2) key stakeholders (policymakers, senior and middle‐level managers, and disasters management experts) with ≥1 year of relevant experience in disability or disasters programs.

Purposive and snowball sampling continued until data saturation. Participants also recommended other eligible individuals. Data were collected using semi‐structured interview guides. Questions for people with disabilities addressed access to health services (e.g., “Did you have adequate access to health services during the Arbaeen pilgrimage?”). Policymakers were asked about inter‐organizational collaboration, health services planning, and procurement of medicines/equipment. The full interview guide is presented in the supplementary file (Table 1).

**TABLE 1:** The interview guide for identifying the challenges and solutions of providing health services to people with disabilities during mass gatherings.

People with disabilities	Health experts and managers
Does the delivery of health services to individuals with disabilities during mass gatherings meet their unique needs and circumstances? What challenges do people with disabilities face during mass gatherings regarding receiving health services? What are the specific challenges that hinder the delivery of health services to individuals with disabilities during the Arbaeen pilgrimage? What principles must be taken into account when delivering health services to individuals with disabilities during large events? Considering the physical conditions of individuals with disabilities and the impact of diseases, what elements must be taken into account when delivering health services to those individuals during large events? Taking into consideration your experience as well as the circumstances surrounding individuals with disabilities and their participation in large gatherings, along with the characteristics of such events regarding potential trauma, the risk of epidemics, and other factors, what recommendations can you provide to enhance the delivery of health services for people with disabilities during the Arbaeen pilgrimage? What are your priority areas for receiving health services? What services, tools, and equipment are needed, and where should they be available?	What challenges are recognized by the authorities concerning the health of people with disabilities during mass gatherings? What challenges impede the delivery of health services to individuals with disabilities, especially during the Arbaeen pilgrimage? What resources are key in providing health services to people with disabilities during mass gatherings? What kind of health services do you think should be available for attendees with disabilities at major events? Are there any specific programs available to offer health services to individuals with disabilities during large events? Considering the physical conditions of individuals with disabilities and the impact of diseases, what elements must be taken into account when delivering health services to people with disabilities during large events? What types of collaboration should be established with other aid organizations to deliver health services to individuals with disabilities? What are the unique characteristics of delivering health services to individuals with disabilities in Iran during large‐scale events? From your experience, what makes healthcare access hardest for pilgrims with disabilities? What initiatives should be implemented to oversee the well‐being of individuals with disabilities? What essential health equipment, services, and specialists are needed for attendees with disabilities? What medications and equipment are required for individuals with disabilities during large events?

### 2.3 Data Collection

Semi‐structured interviews were conducted from May 6 to August 22, 2023. Initial interviews guided the subsequent ones. The study aims were explained, and informed consent was obtained. For people with disabilities, interviews took place in accessible locations. Managers and policymakers were held in their offices following official coordination. All participants were assured of confidentiality. Face‐to‐face interviews began with open‐ended questions then progressed to in‐depth topics.

### 2.4 Data Analysis

Data were analyzed using Lundman and Graneheim's conventional content analysis framework [17]. Initial codes were grouped into subcategories and the main categories. Themes were reviewed by two researchers and validated through member checking with participants.

### 2.5 Rigor

In order to ensure data validity, four criteria of Lincoln and Guba (1985), including credibility, transferability, dependability, and confirmability, were used in this study [18, 19].

To ensure credibility, we employed several strategies. First, we shared a summary of our preliminary findings with 15 of participants to verify the accuracy of our interpretations. Second, the researchers dedicated substantial time to data collection and analysis, ensuring prolong engagement with the data and the research environment. In this study, to ensure transferability, a detailed description of the research context, participant characteristics, selection methods, and data analysis procedures is provided in the relevant sections. To ensure methodological rigor, the team documented the research process in detail, enabling transparent replication and comprehensive understanding of all study phases. The lead researcher and two team members independently reviewed selected interviews, codes, and categories through peer debriefing. Constant data comparison ensured analytical rigor and category validation.

## 3 Results

A total of 26 individuals participated: 15 managers and policymakers (57.7%) and 11 people with disabilities (42.3%). Demographic characteristics are shown in Table 2.

**TABLE 2:** The demographic characteristics of the study participants.

Demographic variables		Managers and experts (*N* (%))	People with disability (*N* (%))
Gender	Male	13 (86.66)	8 (72.72)
	Female	2 (13.33)	3 (27.27)
Age (years)	20–30	5 (33.33)	2 (18.18)
	31–40	1 (6.66)	4 (36.36)
	41–50	2 (13.33)	4 (36.36)
	51–60	6 (40)	1 (9.09)
	>61	1 (6.66)	—
Education level	Under diploma	—	4 (36.36)
	Diploma	—	5 (45.45)
	BSc	8 (53.33)	2 (18.18)
	MSc	4 (26.66)	—
	PhD	2 (13.33)	—
	Specialist	1 (6.66)	—
Work experience (years)	1–10	5 (33.33)	2 (18.18)
	11–20	1 (6.66)	4 (36.36)
	>20	9 (60)	5 (45.45)
Type of disability	Physical disability	—	9 (81.81)
	Visual impairment/Blindness	—	2 (18.18)
	Mental/Psychological disability	—	0 (0)
Category of participants		15 (57.7)	11 (42.3)

**FIGURE 1:**
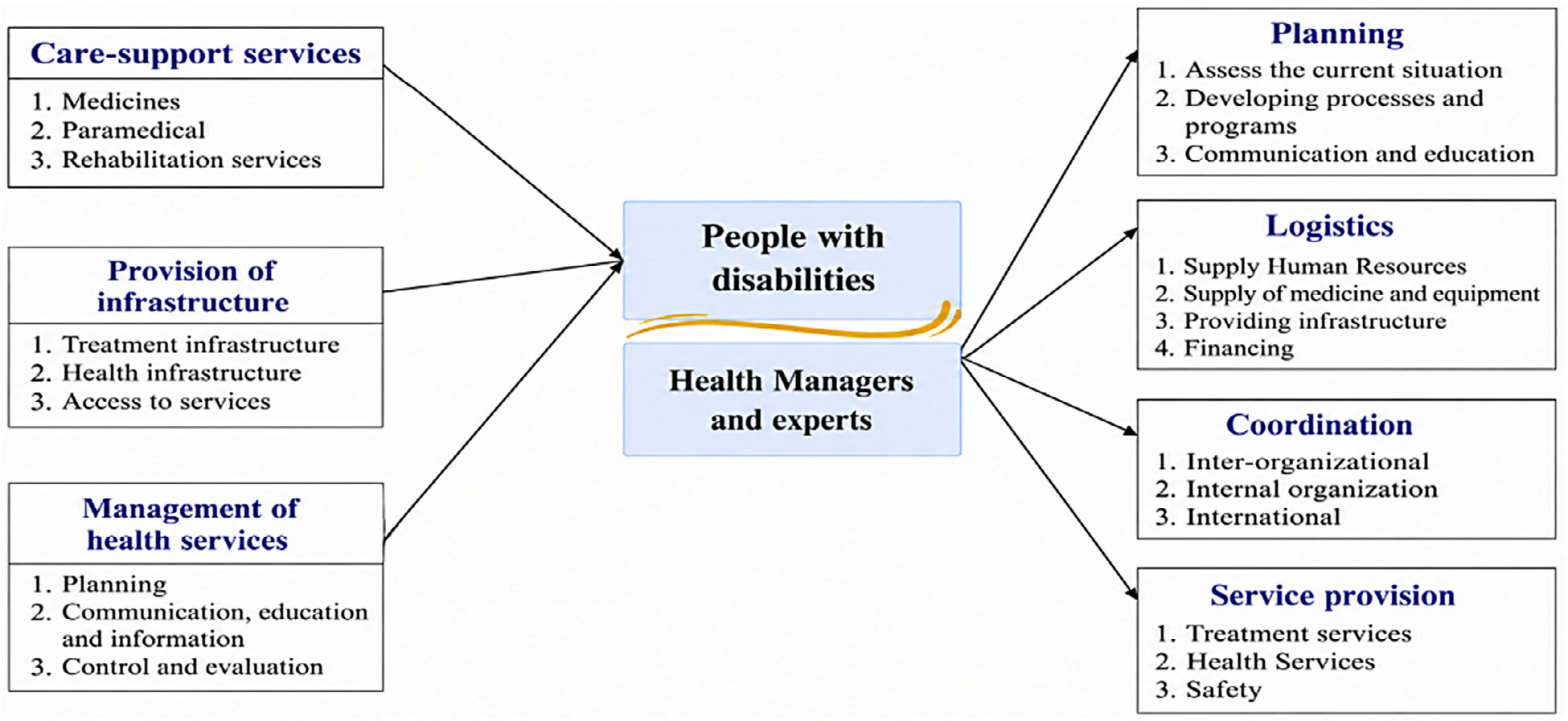
Categories and subcategories resulting from identifying the challenges and proposed solutions in providing health services to people with disabilities during mass gatherings from the perspective of people with disabilities and health system experts and managers.

Initial analysis yielded 475 codes, which reduced to 294 after removing duplicates. Under the supervision of Z.E. and A.D., codes were grouped into subcategories and then 7 main categories with 23 subcategories (Figure 1). The conceptual framework summarizes the key challenges and solutions identified by both groups.

## 4 Responders

### 4.1 Challenges in receiving health services in mass gatherings

Challenges fell into three categories: (1) care‐support services, (2) logistics/infrastructure, and (3) health services management.

#### 4.1.1 Care Support Services

Challenges included medication access, specialized professionals, provider awareness, physiotherapy, social work services, essential equipment, dietary needs, and rehabilitation services:

I use a prosthesis, but this need is often overlooked in healthcare service, possibly due to staff shortages or lack of training. We are largely deprived of rehabilitation services during the Arbaeen pilgrimage.

#### 4.1.2 Logistics and Infrastructure for Health Services Provision

Barrier included failure to address therapeutic needs, inadequate infrastructure, lack of disability‐specific services, and limited access to suitable medical equipment:

Access Ramps to Hospitals and Mobile Medical Centers Are Not Available. Most Services Cater to Healthy Individuals; and Equipment Often Inaccessible to Those With Disabilities.

I have a speech disorder. During the Arbaeen pilgrimage, there was no way to communicate With the medical personnel, which prevented effective communication.

## 5 Health System Experts and Managers

### 5.1 Challenges Experienced by Experts and Health Managers in Providing Health Services to People With Disabilities

Challenges included insufficient qualified staff, limited equipment, and inaccessible facilities in high‐traffic zones, limited insurance coverage, and inadequate funding for infrastructure:

We lack of specialized personnel and insufficient medical supplies for the underlying health conditions of individuals with disabilities. Lack of crucial health infrastructure in Iraq also caused complications.

## 6 Discussion

Findings revealed significant barriers faced by both service providers and recipients. Managers and policymakers faced challenges in planning, logistics, coordination, and provision. People with disabilities faced challenges in care‐support services, infrastructure provision, and health services management. Existing planning is more ad hoc and revision with disability community participation. Planners must assess needs and allocate resources on the basis of real‐world data [20]. Koski et al. also emphasized coordinated preparation and multi‐sectoral collaboration [21].

Logistics challenges (human resources, supplies, infrastructure, and financial) align with prior research [22, 23]. Quality care requires expertise, not just in terms of more staff [21], but also special protocols, training, and optimized human are essential for people with disabilities. Insufficient coordination (intra, inter, and international) was another challenge. Inter‐agency agreements clarify responsibilities [24]. Scarce medical resources require international collaboration [25]. Multi‐sectoral coordination frameworks can prevent inequitable resource allocation. Challenges included financial difficulties and lack of provider's expertise [26], challenges are exacerbated in mass gatherings. Iraq's prehospital emergency systems face staff and ambulance shortages [22]. Needs‐based strategies can improve access [27].

Infrastructure challenges included treatment facilities, health infrastructure, and access to services. Population displacement creates health disparities requiring strategic planning [28]. Dong et al. emphasized for strengthening emergency teams at mass gatherings due to high crowd density and environmental risks [29]. Accessibility accommodations (e.g., ramps and wheelchair) at Arbeen pilgrimage are inconsistent and inadequate.

Health services management challenges included planning, communication, education, and evaluation. Resources demand depended on event duration, climate, crowd size, and context [30]. A coherent disaster response plan with clear roles and communication protocols is necessary [31]. Similar to Hajj health guidelines [32], proactive measures, including (1) pre‐participation assessments, (2) targeted vaccination, and (3) health education, are essential [33]. Dassah et al. emphasized in‐service training to improve communication with patients with disabilities [34].

Action is needed at two levels: short term and long term. Short‐term action enhanced planning, institutional coordination, and cross‐border cooperation, whereas long‐term action co‐designed services with disabled communities and embedded disability‐responsive frameworks into crisis management policies. Future studies should evaluate the effectiveness and cost‐effectiveness of these solutions.

Table 3 presents practical recommendation for improving health service delivery to people with disabilities at mass gatherings.

**TABLE 3:** Recommendation for improving health service delivery to people with disabilities at mass gatherings.

Row	Challenges	Solutions
1	**Planning**	Needs assessment, pre/during/post‐event planning, specialized health records, disability inclusive protocols, training programs, and infectious disease forecasting
2	**Logistics**	Recruit experienced disability care providers and supply essential medications on preliminary needs
3	**Coordination**	MOUs with insurance, coordinate among Red Crescent, Welfare Organization, and Ministry of Health
4	**Services provision**	Nutritional support, clear treatment information, priority based on needs severity, and accessible exam areas
5	**Care support**	Sign language interpreters, psychological counseling, rehabilitation programs, and accessible service centers
6	**Infrastructure**	Separate service channels, designated zones for disability units, ramps, tactile pathways, and adapted sanitation
7	**Management health services**	Standardized guidelines, specialized training for staff, expert medical teams, and disability‐adapted education

## 7 Strengths and Limitations of the Study

**Strengths**: Diverse participant sample (policymakers, managers, and people with disabilities) allowing provider and recipient perspectives.

**Limitations: (**1) Few female participants (*n* = 3) limit gender analysis. (2) Low disability participation due to inaccessible infrastructure and limited support services. (3) Non‐probabilistic sampling prevents generalizability, and findings offer context‐specific insights.

## 8 Suggestions for Future Research

The research team recommends future studies that directly engage diverse participants with disabilities (including varying impairment types and socioeconomic backgrounds) who have attended mass events like the Arbaeen pilgrimage. This may include cooperative approaches like community‐based participatory research (CBPR) to address recruitment accessibility challenges. Extensive surveys or mixed‐methods research could measure the frequency of reported issues (e.g., percentage of PWDs who cannot access emergency care) to guide resource allocation priorities.

## 9 Conclusion

Mass gatherings, such as the Arbaeen pilgrimage, pose considerable challenges in providing equitable healthcare access for individuals with disabilities. Addressing these challenges necessitates compliance with established health policy standards, including universal health coverage (UHC), inclusive design principles, and emergency preparedness protocols that are inclusive of disabilities. In order to ensure equity in access to health services, this study highlighted two key policy strategies: (1) the ongoing and meaningful participation of people with disabilities in designing, planning, and implementation health programs, and (2) multi‐sectoral collaboration among health policymakers and relevant institutions to effectively integrate the needs of people with disabilities into national and local mass gathering management programs.

## Author Contributions

Zahra Eskandari and Arezoo Dehghani contributed to the conception and design of the research. Gholamreza Masoumi drafted the article. Fateme Behmenshpour and Sajjad Anami conducted the analysis. Arezoo Dehghani and Gholamreza Masoumi supervised the analysis. All authors contributed to manuscript revision, approved the submitted version, and agreed to be accountable for the work.

## Funding

The authors have nothing to report.

## Disclosure

All authors have reviewed the final version of the manuscript and approved it for submission. All authors agree to be accountable for all aspects of the work. The authors have presented an accurate account of the work performed and an objective discussion of its significance. All sources of information and data used in this research have been properly cited.

## Ethics Statement

This study received ethical approval from the Ethics Committee of Alborz University of Medical Sciences (Code: IR.ABZUMS.REC.1402.031). Participants were informed that their participation was entirely voluntary, and that they could withdraw at any time without any consequences.

## Consent

Informed consent was obtained from all the participants for interview and recording, both orally and writing. Participants were assured of the confidentiality of their information.

## Conflicts of Interest

The authors declare no conflicts of interest.

## Data Availability

The datasets used and/or analyzed during the current study are available from the corresponding author on reasonable request.
